# Dipstick Proteinuria and Hematuria as Triggers for Manual Microscopic Review in Nephrology Patients

**DOI:** 10.3390/jcm14134522

**Published:** 2025-06-26

**Authors:** Priscila Aparecida Correa Freitas, Yasmini Dandara Silva da Silva, José Antonio Tesser Poloni, Francisco José Veríssimo Veronese, Luiz Felipe Santos Gonçalves

**Affiliations:** 1 Hospital de Clínicas de Porto Alegre, Porto Alegre 90035903, Brazil; fveronese@hcpa.edu.br; 2 Universidade Federal do Rio Grande do Sul, Porto Alegre 90035003, Brazil; yasmini.dandara@gmail.com; 3 Controllab—Quality Control for Laboratories, Rio de Janeiro 20911442, Brazil; joseatpoloni@gmail.com

**Keywords:** urinalysis, nephropathy, urinary sediment, microscopy

## Abstract

**Background/Objectives:** Automation improves efficiency in laboratory workflow but may fail to detect clinically relevant abnormalities in patients with nephropathy. This study aimed to identify dipstick parameters associated with nephropathy-related sediment findings and to propose practical criteria to guide manual microscopy review based on these associations. **Methods:** Urine samples from in- and outpatients, primarily from the nephrology unit, were collected at a university hospital from July 2022 to September 2023. Samples were analyzed within two hours using LabUMat 2 and UriSed 3 analyzers. Manual microscopy was performed on all specimens by two experienced technicians. Sediments were classified as suggestive or not of nephropathy based on hematuria with dysmorphism, hyaline and pathological casts, lipiduria, or renal tubular epithelial cells. **Results:** Of 503 samples, 146 (29%) showed sediment findings indicative of nephropathy, which were significantly associated with dipstick positivity for protein and blood. Among nephropathy samples, 71.2% had protein ≥1+ or blood ≥2+. Using this combination as a criterion for manual sediment review yielded a sensitivity of 71.2%, a specificity of 73.9%, and a 3.84-fold increased relative risk of detecting nephropathy-related elements (*p* < 0.001). The criteria performed best among nephrology outpatients, with sensitivity of 79.5%, specificity of 63.9%, and relative risk of 3.91 (*p* < 0.001). **Conclusions:** Dipstick protein ≥1+ or blood ≥2+ helps identify patients who may benefit from manual sediment review, supporting diagnostic accuracy in nephropathy. Each institution should define its criteria based on patient profile, analytical methods, and workflow.

## 1. Introduction

Routine urine microscopy performed by laboratory technicians presents several challenges, such as handling a high volume of samples, the time-consuming nature of the test, and the fact that urine samples should ideally be assessed within two hours of collection, unless a preservative is used [1,2].

The introduction of automated urinalysis technology in 1985 [3] led to its widespread adoption. In addition to its significant high-throughput advantages, automated analysis standardizes the parameters for microscope measurements and can seamlessly integrate with computerized clinical information systems for data transmission, storage, and analysis, minimizing the risk of transcription errors [4]. Moreover, with advancements in technology, valuable microscopic techniques, such as phase contrast and digital microscopy, have already been implemented, enhancing the quality and precision of the analysis [5]. While automation enhances efficiency, it may not always capture subtle or complex abnormalities that are crucial for accurate diagnosis in such patients. Therefore, careful consideration is required when interpreting results, particularly in high-risk populations, to ensure that critical findings are not overlooked [6] particularly in patients with kidney disease [7,8]. Manual urine sediment examination provides a level of detail that automated systems often fail to achieve [9,10]. Integrating automated analysis with targeted manual review for elements that are challenging for automation has improved both diagnostic accuracy and reproducibility while streamlining processing times [11,12]. However, the decision to perform a manual review is typically made by the laboratory technician, highlighting the need for well-defined, institution-specific criteria to guide this process. Establishing such criteria is essential to ensure consistency and optimize the diagnostic accuracy and efficiency of urinalysis.

The transition of urinalysis from nephrology clinics to large-scale automated laboratories has raised concerns about the potential for false-negative results. Routine urinalysis typically involves a two-step process, beginning with a physicochemical assessment using a dipstick [13]. While dipstick analysis alone is insufficient for accurately evaluating renal patients [14], its combination with urine sediment analysis can yield critical insights into the specific renal compartment affected [10]. Given the impracticality of performing manual sediment reviews on all samples in high-throughput laboratory workflows, and the need to improve the detection of nephropathy-related abnormalities, this study aimed to identify dipstick parameters associated with such findings and to propose practical criteria to guide manual microscopic review in a university hospital setting.

## 2. Materials and Methods

The detailed procedures for this study were carried out as previously described by the authors [6].

A total of 503 urine specimens were obtained from inpatients and outpatients at the Clinical Laboratory of a University Hospital in Southern Brazil between July 2022 and September 2023. Analyses were performed twice weekly, selecting 10–20 urine specimens per day through convenience sampling, corresponding to the days when the study was conducted, with a predominance of patients from the nephrology outpatient clinic, which constituted the primary source of samples included in the study. However, within this available subset, samples were randomly selected in an attempt to minimize sampling bias. Specimens were freshly collected in sterile containers, each containing 10 mL of urine, and were analyzed within 2 h using the LabUMat 2 (a chemical analyzer employing LabStrip U11 Plus test strips) and Urised 3 instruments (a sediment analyzer with digital microscopy technology), which are interconnected (77 Elektronika Kft., Budapest, Hungary). The Labstrip U11 Plus determines various chemical parameters according to specific ranges: bilirubin (negative if <0.5 mg/dL); urobilinogen (normal if <2 mg/dL); ketones (negative if <5 mg/dL); ascorbic acid (negative if <20 mg/dL); glucose (normal if <25 mg/dL); protein (negative if <15 mg/dL/trace if 15–29 mg/dL/1+ if 30–99 mg/dL/2+ if 100–499 mg/dL/3+ if ≥500 mg/dL); blood/erythrocytes (negative if <5/μL/+1 if 5–49/μL/2+ if 50–299/μL/3+ if ≥300/μL); pH (5–9); nitrite (negative if absent); and leukocytes (negative if <25/μL).

In the daily laboratory’s routine, specimens are sent to manual microscopic review for further investigation at the discretion of the technologists, following standard operating procedures. Currently, only 10% of all urine samples received each day are reviewed. However, in this study, all selected specimens underwent manual microscopic examinations conducted by two well-trained technologists using the same slide for both technologists’ analyses, employing phase-contrast light microscopy (Olympus BX40, Olympus, Tokyo, Japan). Upon completion of the individual assessments, the findings were reviewed and compared. In cases of qualitative or quantitative discrepancies, the samples were re-examined jointly until a consensus was reached. Quantitative data were reported as the average of the values obtained by the two observers. Although the technologists were selected based on their expertise, they underwent an “interobserver variability calibration” prior to data collection. For this purpose, a set of urine samples exhibiting a range of sediment alterations was reviewed qualitatively and quantitatively in weekly consensus meetings over a 1-month period. This preparatory phase ensured alignment in interpretation and strengthened consistency across independent analyses.

All samples were centrifuged at 400× *g* for 5 min, and 20 microliters of resuspended sediment were pipetted onto a microscope slide and covered with a 22 × 22 mm coverslip. Low-power field (LPF, ×100 magnification) was used for the analysis of casts and squamous and transitional epithelial cells, while high-power field (HPF, ×400 magnification) was employed for the examination of red blood cells, white blood cells, non-squamous and transitional epithelial cells, oval fat bodies, yeasts, and crystals. All urinary sediment particles were analyzed in 10 fields at each magnification. Red blood cells and white blood cells were expressed as <6.4/μL or <8.3/μL if <1 cells/HPF, or >547.4/μL or >557.1 if >100 cells/HPF, respectively.

The sediment results were classified into two different categories based solely on sediment findings: a pattern suggestive of nephropathy, defined by the presence of hematuria with dysmorphism (≥23.6 red blood cells/μL or ≥3 red blood cells/HPF), multiple hyaline casts (≥4 particles/LPF or ≥1.9 particles/μL), >1 pathological cast (granular, epithelial, red blood cells, waxy, or white blood cells casts), >1 element of lipiduria (fatty casts or oval fat bodies), or >1 renal tubular epithelial cells; and absence of nephropathy, characterized by the lack of these abnormalities, although minor alterations such as isolated hematuria without dysmorphism, leukocyturia, or occasional hyaline casts (<4 particles/LPF or <1.9 particles/μL) could be presented.

Data were analyzed using PASW Statistics for Windows version 29.0 (SPSS, Inc., USA). Categorical variables were expressed as percentages (%) and proportions. Differences in proportions between groups were assessed using the chi-square test. The sensitivity (SN), specificity (SP), and accuracy [(true positives + true negatives)/N = 503] were calculated for the chemical parameters to detect patterns indicative of nephropathy in urine. Poisson regression analysis was conducted to model the association between dipstick chemical parameters and nephropathy-related sediment findings, estimating relative risks and 95% confidence intervals. Model assumptions, including overdispersion, were verified. A significance level of *p* < 0.05 was adopted for all analyses.

The study was conducted in accordance with the Declaration of Helsinki and was approved by the Ethics Committee of Hospital de Clínicas de Porto Alegre (CAAE: 34484720.4.0000.5327; approval date: 11 August 2020). Ethical approval covered the use of anonymized data and discarded urine samples from routine laboratory workflow, with no additional interventions.

## 3. Results

### 3.1. Patient Characteristics

Among the 503 urine samples analyzed, 263 (52.3%) were obtained from nephrology outpatients, while 248 were from patients diagnosed with kidney diseases, including chronic kidney disease, acute renal failure, or glomerulopathy, with or without proteinuria. Of these, 146 samples exhibited sediment abnormalities indicative of nephropathy, as identified through the reference manual analysis performed in this study. The median age of the patients was 56 years [interquartile range, 44–64], and 53.7% were male.

To establish optimal criteria for manual sediment review in routine urinalysis, we first cross the dipstick results for bilirubin, urobilinogen, ketones, nitrite, leukocytes, glucose, protein, and blood/erythrocytes (positive or negative) with the presence of nephropathy-related findings using the chi-square test (Supplementary Table S1). In this analysis, only protein and blood demonstrated statistically significant associations with nephropathy findings (*p* < 0.001, Table 1).

### 3.2. Dipstick Blood and Protein as Triggers for Sediment Review

Subsequent analyses focused exclusively on these two chemical markers. Table 1 displays the corresponding percentages. Protein levels showed significant differences across all quantification categories (0 to +3). In contrast, blood levels demonstrated significant differences only in the 0, 2+, and 3+ categories. The absence of both protein and blood was most frequently observed in samples lacking nephropathy-related characteristics.

The next step involved evaluating the utility of dipstick protein and blood levels, either individually or in combination, as potential criteria for manual sediment review. Table 2 summarizes the distribution of these markers among patients presenting nephropathy-related findings in urine sediment. Notably, the combination of protein levels ≥1+ or blood levels ≥2+ was the most frequent, occurring in 71.2% of these patients.

Table 3 presents the diagnostic performance of these potential review criteria, excluding protein ≥3+ and blood ≥3+ due to their low prevalence in the nephropathy sample (<15%). The combination of protein ≥1+ or blood ≥2+ showed the most balanced sensitivity (71.2%) and specificity (73.9%). Moreover, this combined criterion was associated with a 3.84-fold increased risk of detecting nephropathy-related elements in urine sediment (RR: 3.84; *p* < 0.001).

Table 4 summarizes the diagnostic performance of protein (≥1+) or blood (≥2+) as review criteria for detecting nephropathy-associated findings, stratified by patient origin. For nephrology outpatients the criteria yielded the highest SN (79.5%) but lower SP (63.9%), along with a 3.91-fold increase in relative risk (RR) for identifying pathological sediment findings (*p* < 0.001). Among other outpatients, the criteria were associated with the highest SP (86.6%) and RR (5.03-fold), but the lowest SN (58.3%). No statistical association was observed among inpatients or emergency department patients. Furthermore, these criteria showed the strongest association with nephropathy-related urinary sediment components in nephrology outpatients (131/263, 49.8%; *p* = 0.037, Pearson’s chi-square test).

## 4. Discussion

In urinalysis, it is well established that chemical analysis alone leads to significant diagnostic losses, necessitating both semi-quantitative dipstick assessment and microscopic sediment analysis for comprehensive evaluation [15]. Automated urinalysis integrates both chemical and sediment analysis, improving laboratory efficiency but introducing diagnostic challenges in renal disease by potentially overlooking critical urinary sediment elements. Despite the role of manual microscopy as a confirmatory tool, the high sample volumes, productivity pressures, and limited clinical data frequently compromise diagnostic accuracy in clinical laboratories [16,17].

The authors’ previous study assessed the diagnostic accuracy of urinalysis in a clinical laboratory using an automated microscopy system, with images analyzed both by the system and laboratory technologists, in patients with (N = 248) and without (N = 255) kidney disease. The study revealed low SN for detecting pathological urinary elements (casts, lipiduria, renal tubular cells) in both groups. However, manual sediment review significantly improved the identification of nephropathy-related elements. These findings underscore the critical role of traditional microscopy in kidney disease diagnosis, improving accuracy and potentially impacting clinical management [6].

Despite this, as laboratory staff, we acknowledge that technologist screening is challenging, not only due to the high volume of daily urinalysis but also because of the number and/or quality of images produced by the automated system, as well as the use of unclear or overly permissive criteria for manual review, intended to prevent overburdening the manual review process with excessive FP samples. Therefore, in the current study, in order to simplify the rules for manual review, we proposed a review criterion for automated urine sediment analyzers based on an optical system without flow cytometry (Urised 3 PRO instrument), using dipstick results (LabUmat 2 instrument). We focus on enhancing the urinalysis performance with a criterion validated to predict pathologic elements usually found in patients with nephropathy, evaluated with a reference analysis [6]. Accordingly, we did not set our review criteria for isomorphic red blood cells or white blood cells counts, nor to squamous epithelial cells, yeast, sperm or crystals.

Thereby, protein ≥1+ (or ≥30–99 mg/dL) and blood ≥2+ (or ≥50–299/μL) on dipstick results were significantly associated with the presence of nephropathy-related elements in sediment. Among patients without nephropathy findings, 82.1% exhibited no proteinuria, and 88.7% had absent or only 1+ of blood (Table 1). Applying this combination as a review criterion would have selected 104 out of 146 patients with pathological sediment patterns for manual microscopy, irrespective of the laboratory technologist’s analysis, thereby increasing the likelihood of accurately reporting critical elements (Table 2).

While some studies have proposed review criteria based on dipstick results combined with flow cytometry flags [12,15] or relying solely on flow cytometry data [18,19,20,21], Du et al. (2015) [22] conducted an evaluation integrating dry-chemistry analysis with three different digital imaging systems (IQ200, AVE766, and US2026). In their study, the optimal review criteria included the presence of blood ≥2+ or protein ≥2+ on the dry-chemistry test; a red blood cells count exceeding twice the upper reference limit in the formed element analysis; discordant white blood cells results between the two detection methods; or casts above the reference threshold in sediment analysis. Using these criteria, the average FP and FN rates were 26.5% and 1.9%, respectively [22]. However, none of the aforementioned studies proposed review criteria based on the presence of nephropathy-associated sediment findings as the primary indicator for manual microscopy, as done in our study, which limits the possibility of direct comparisons.

Additionally, the presence of urinary protein ≥1+ or blood ≥2+ was associated with a 3.84-fold increased RR of a nephropathy-pattern sediment in the overall sample (Table 3). Although this criterion yielded a balanced SN and SP of 71.2% and 73.9%, respectively, it demonstrated the highest FP (N = 93 or 18.5%), but the lowest FN (N = 42 or 8.3%) among the evaluated criteria. High-quality microscopic review protocols should prioritize enhanced SN to reduce clinically significant omissions while maintaining an acceptable overall review rate. However, integrating these two principles can be challenging, depending on the institutional profile. In this context, Gai et al. have proposed performing microscopic reviews on all samples with known or suspected renal diseases without implementing a specific algorithm [23].

To further investigate the nature of FP cases, we reviewed the clinical records of the 93 patients misclassified by our review criteria. Remarkably, 63 of them had documented renal disease: 58 with chronic kidney disease (CKD), 3 with acute kidney injury (AKI), and 2 with glomerulopathies presenting with proteinuria. Notably, all FP cases met the review criteria due to the presence of proteinuria. CKD may present solely with proteinuria, particularly in its early stages, without accompanying sediment abnormalities [24]. Likewise, in AKI, the absence of sediment alterations does not preclude diagnosis. According to KDIGO criteria, AKI is defined and staged based on changes in serum creatinine and urine output, independent of microscopic findings [25].

Despite the clinical relevance of hematuria and proteinuria detected by dipstick testing in urinalysis, it is well established that various non-nephrological conditions can also yield such findings. For example, non-glomerular hematuria may occur in individuals with hematologic disorders, nephrolithiasis, malignancies, urinary tract infections, or benign prostatic hyperplasia. Similarly, transient proteinuria may arise in response to non-pathological or systemic conditions, such as dehydration, exposure to extreme temperatures, emotional stress, fever, acute illness, inflammation, vaginal secretions, intense physical exertion, or orthostatic changes (postural proteinuria) [26].

Finally, additional limitations must be considered when applying the review criteria proposed in this study. The SN of dipsticks for detecting hematuria ranges from 95% to 100%, while SP varies between 65% and 93% [13]. Multiple factors may influence test accuracy. Dipsticks detect the peroxidase activity of red blood cells; however, the presence of other peroxidase-containing substances in urine can result in FP results. These include myoglobin (released from muscle injury), free hemoglobin (from intravascular hemolysis), semen, certain bacterial species (Enterobacteriaceae, Staphylococci, and Streptococci), blood contamination (from menstruation and improper specimen collection), and specific vegetables. Conversely, FN results are often associated with the presence of ascorbic acid, which inhibits the peroxidase reaction [13,26,27]. Regarding proteinuria, dipstick testing shows high SP, but low SN, as the method is more responsive to albumin than to tubular proteins or light chain immunoglobulins [14,26]. Furthermore, FP results may occur in highly buffered alkaline urine (pH 9), typically caused by alkaline medications or stale urine [1].

Additionally, the assessment of the review criteria by patient origin (Table 4) was not conclusive for inpatients due to the limited sample size (N = 47). In contrast, for outpatients, both groups demonstrated statistically significant associations with nephropathy-related findings in the Poisson regression. Among nephrology outpatients, the RR was 3.91 [95% IC: 2.43–6.29], with greater precision attributable to a larger sample size (N = 263) and higher number of exposed cases (N = 131). For other outpatient departments, the RR was higher (5.03 [95% CI: 2.85–8.88]), although the wider confidence interval reflects increased uncertainty due to a smaller number of exposed individuals (N = 41 out of 193). Moreover, the application of the review criteria would select approximately half of nephrology outpatients (49.8%) for manual review, resulting in a higher FP rate (24.7%) compared to the total 503 sample (18.5%), but with a reduction in FN (6.5% vs. 8.3%). For other outpatients, only 21.8% would be reviewed, with half of these representing FP cases, while FN rates remained similar (7.8%).

Implementing the proposed microscopic review criterion would substantially modify the current workflow in our laboratory. At present, we process approximately 850 urine samples per week, with manual microscopic reviews performed on about 10% of them. Adoption of the new criterion would result in a 4-fold increase in the number of manual reviews compared to current practice. However, if applied selectively to nephrology outpatients and hospitalized or emergency inpatients, the increase would be approximately 2.9-fold. When restricted exclusively to nephrology outpatients, the criterion would lead to only a 1.6-fold increase in manual reviews. This targeted implementation could improve the detection of nephropathy-related sediment abnormalities in a pre-selected population, thereby potentially enhancing diagnostic accuracy and patient outcomes.

This study presents notable strengths and limitations. A key strength is the evaluation of urinalysis review criteria based on dipstick results in a clinically relevant population of patients with confirmed kidney disease followed at a tertiary care center. To the best of our knowledge, this is the first study to propose review criteria derived from nephropathy-associated findings in urinary sediment, identified through a blinded reference analysis conducted by experienced microscopists.

However, several limitations must be acknowledged. First, despite efforts to randomize within the available samples, the absence of true randomization in patient selection introduces potential selection bias, which may affect the representativeness of the sample. Second, the predominance of samples from nephrology units could lead to an overestimation of the criteria’s performance compared to general or asymptomatic populations. Third, the single-center design limits the generalizability of the findings, and external validation in multicenter and more heterogeneous settings is needed. Fourth, the study did not control for potential confounding factors, such as medications or comorbidities, that may influence dipstick results. Finally, the lack of access to raw sediment data generated by the analyzer prior to manual verification precluded the assessment or development of review criteria combining dipstick results from the LabUMat 2 system with unverified sediment findings from the Urised 3 PRO system.

## 5. Conclusions

In conclusion, review criteria based on the presence of protein and blood on dipstick testing may assist in the screening of urine samples by reducing the likelihood of FN results in urinary sediment analysis. However, it is essential that each laboratory establish its own review criteria, taking into account differences in specimen origin, analytical methodologies, and workflow. Moreover, any proposed criteria should undergo continuous validation and periodic updates to enhance the reliability of automated urinalysis. Finally, given the simplicity and clinical value of urinalysis, institutions should be encouraged to promote ongoing microscopy training for laboratory technologists, as the effectiveness of any review criterion ultimately depends on the quality of sediment examination.

## Figures and Tables

**Table 1:** Distribution of protein and blood levels based on nephropathy-related findings in sediment analysis.

		*Protein 0*	*Protein 1+*	*Protein 2+*	*Protein 3+*	*p*-Value
**Nephropathy**	**Absent**	293 (82.1%)	32 (9.0%)	29 (8.1%)	3 (0.8%)	<0.001 *
	**Present**	69 (47.3%)	32 (21.9%)	38 (26.0%)	7 (4.8%)	
		***Blood 0***	***Blood 1+***	***Blood 2+***	***Blood 3+***	
	**Absent**	244 (68.3%)	73 (20.4%)	31 (8.7%)	9 (2.5%)	<0.001 ^#^
	**Present**	63 (43.2%)	31 (21.2%)	31 (21.2%)	21 (14.4%)	

Data are presented as N—sample size (%), with percentages calculated and displayed by rows. * *p* < 0.001 for all categories, determined by the chi-square test. # *p* < 0.001 for 0, 2+, and 3+ categories, determined by the chi-square test.

**Table 2:** Positivity rates of potential review criteria in urine samples with nephropathy-related findings (N = 146).

***Protein (≥1+)***	77/146 (52.7%)
***Protein (≥2+)***	44/146 (30.1%)
***Protein (≥3+)***	7/146 (4.8%)
***Blood (≥2+)***	52/146 (35.6%)
***Blood (≥3+)***	21/146 (14.4%)
***Protein (≥1) or blood (≥2+)***	104/146 (71.2%)
***Protein (≥1) or blood (≥3+)***	84/146 (57.5%)

Data are presented as N—sample size (%).

**Table 3:** Diagnostic performance of different review criteria for nephropathy-related findings in the entire sample (N = 503).

	Total	TP	FP	TN	FN	SN	SP	Accuracy	RR [95% CI]	*p*-Value
***Protein (≥1+)***	141	77	64	293	69	52.7%	82.1%	73.6%	2.62 [1.89–3.63]	<0.001
***Protein (≥2+)***	87	45	32	325	101	30.8%	91.0%	73.6%	2.98 [2.21–4.00]	<0.001
***Blood (≥2+)***	92	52	40	317	94	35.6%	88.8%	73.3%	2.43 [1.75–3.40]	<0.001
***Protein (≥1+) or blood (≥2+)***	**197**	**104**	**93**	**264**	**42**	**71.2%**	**73.9%**	**73.2%**	**3.84 [2.82–5.25]**	**<0.001**
***Protein (≥1+) or blood (≥3+)***	154	84	70	287	62	57.5%	80.4%	73.8%	3.07 [2.35–4.01]	<0.001

Total: Number of patients with nephropathy-related findings. Data are presented as N (sample size) for TP (True Positive), FP (False Positive), TN (True Negative), and FN (False Negative); SN (Sensitivity); SP (Specificity); RR (Relative Risk); CI (confidence interval); and *p*-value, derived from Poisson regression.

**Table 4:** Diagnostic performance of protein (≥1+) or blood (≥2+) for nephropathy-related findings according to the patient’s origin.

	Total	TP	FP	TN	FN	SN	SP	Accuracy	RR [95% CI]	*p*-Value
***Inpatients/Emergency (N = 47)***	24	17	7	13	10	62.9%	65.0%	63.8%	1.63 [0.96–2.77]	0.72
***Nephrology outpatients (N = 263)***	131	66	65	115	17	79.5%	63.9%	68.8%	3.91 [2.43–6.29]	<0.001
***Other outpatients*** ***(N = 193)***	42	21	21	136	15	58.3%	86.6%	81.3%	5.03 [2.85–8.88]	<0.001

Total: Number of patients with nephropathy-related findings. Data are presented as N (sample size) for TP (True positive), FP (False positive), TN (True negative), and FN (False negative); SN (Sensitivity); SP (Specificity); RR (Relative risk); CI (Confidence interval); and *p*-value are derived from Poisson regression.

## Data Availability

The raw data supporting the conclusions of this article will be made available by the authors on request.

## Supplementary Materials

The following supporting information can be downloaded at: https://www.mdpi.com/article/10.3390/jcm14134522/s1, Table S1: Association between dipstick results other than protein and blood and nephropathy-related findings in sediment analysis.

## Abbreviations

The following abbreviations are used in this manuscript:

TP	True positive
FP	False positive
TN	True negative
FN	False negative
SN	Sensitivity
SP	Specificity
RR	Relative Risk
CI	Confidence interval
AKI	Acute kidney injury
CKD	Chronic kidney disease
