# Supplementary material for: Dipstick Proteinuria and Hematuria as Triggers for Manual Microscopic Review in Nephrology Patients
Source: J Clin Med. 2025 Jun 26;14(13):4522. doi: 10.3390/jcm14134522 (PMC12249889; doi:10.3390/jcm14134522)
Supplement: Supplementary file 1 [file jcm-14-04522-s001.zip › jcm-3643128-supplementary.pdf]

Table S1. Association between dipstick results other than protein and blood and nephropathy-related findings in sediment analysis.

		Nephropathy		p-value
		No	*Yes*	
Bilirubin	**0**	357 (71.4%)	143 (28.6%)	0.061
	**1+**	0 (0.0%)	1 (100.0%)	
	**2+**	0 (0.0%)	1 (100.0%)	
	**3+**	0 (0.0%)	1 (100.0%)	
Urobilinogen	**0**	320 (71.7%)	126 (28.3%)	0.376
	**1+**	31 (62.0%)	19 (38.0%)	
	**2+**	4 (80.0%)	1 (20.0%)	
	**3+**	2 (100.0%)	0 (0.0%)	
Glucose	**0**	305 (70.5%)	125 (29.1%)	0.705
	**1+**	16 (76.2%)	5 (23.8%)	
	**2+**	19 (65.6%)	10 (34.5%)	
	**3+**	13 (81.3%)	3 (18.7%)	
	**4+**	4 (57.1%)	3 (42.9%)	
Ketones	**0**	353 (71.0%)	144 (29.0%)	0.704
	**1+**	3 (60.0%)	2 (40.0%)	
	**2+**	1 (100.0%)	0 (0.0%)	
Nitrite	**Neg**	346 (71.3%)	139 (28.7%)	0.348
	**Pos**	11 (61.1%)	7 (38.9%)	
Leukocytes	**Neg**	242 (70.3%)	102 (29.7%)	0.649
	**Pos**	115 (72.3%)	44 (27.7%)	

Neg: Negative; Pos: Positive. Data are presented as N—sample size (%), with percentages calculated and displayed by rows.
